# Mortality in Cincinnati

**Published:** 1849-08

**Authors:** 


### MORTALITY IN CINCINNATI.

From the 16th of June to the 16th of July, a period of 31 days, the aggregate mortality in that city, from all diseases, was *three thousand six hundred and eighteen*, making, in round numbers, a daily average mortality for the time embraced, of *one hundred and seventeen.*

The weekly mortality for the whole of the above period, is as follows:

| | |
|---|---|
| First three days, to June 18, - - - - - - | 138 |
| First week, to June 25, - - - - - - - | 568 |
| Second week, to July 2, - - - - - - - | 940 |
| Third week, to July 9, - - - - - - - | 1022 |
| Fourth week, to July 16, - - - - - - - | 950 |
| Total, | 3,618 |

In Cincinnati, as elsewhere, the mortality from cholera and other diseases occurring coetaneously with it, has been greatest among the foreign population. This population in that city is nearly one-third of the whole number of its inhabitants, but almost three-fourths of the entire number of deaths is from this class of the population.

